# Author Correction: Neutrophil extracellular traps promote metastasis in gastric cancer patients with postoperative abdominal infectious complications

**DOI:** 10.1038/s41467-025-57805-7

**Published:** 2025-03-10

**Authors:** Xiang Xia, Zizhen Zhang, Chunchao Zhu, Bo Ni, Shuchang Wang, Shuofei Yang, Fengrong Yu, Enhao Zhao, Qing Li, Gang Zhao

**Affiliations:** 1 Department of Gastrointestinal Surgery, Ren Ji Hospital, School of Medicine, Shanghai Jiao Tong University, Shanghai, People’s Republic of China; 2 Department of Vascular Surgery, Ren Ji Hospital, School of Medicine, Shanghai Jiao Tong University, Shanghai, People’s Republic of China; 3 State Key Laboratory of Oncogenes and Related Genes, Shanghai Cancer Institute, Ren Ji Hospital, School of Medicine, Shanghai Jiao Tong University, 200240 Shanghai, People’s Republic of China

Correction to: *Nature Communications* 10.1038/s41467-022-28492-5, published online 23 February 2022

In the version of the article initially published, in Fig. 3c, the representative images (lower panel) of hematoxylin-eosin staining for PM samples should have corresponded properly to the immunofluorescence co-staining images of DAPI, E-cadherin, and N-cadherin, similar to the images shown in the LM part (upper panel) of Fig. 3c. However, in the original version, the representative images of HE staining for PM samples did not match well. Therefore, in this corrected Fig. 3c, we have selected suitable pathological sections of PM for hematoxylin-eosin staining, which now properly correspond to the immunofluorescence co-staining images, as seen in Fig. 1. In Supplementary Fig. 3g, the image for AGS (neutrophils) was incorrect. The amended Supplementary Fig. 3g can be found alongside the online version of the article.

Fig. 1 Original and corrected Fig. 3c

Original Figure
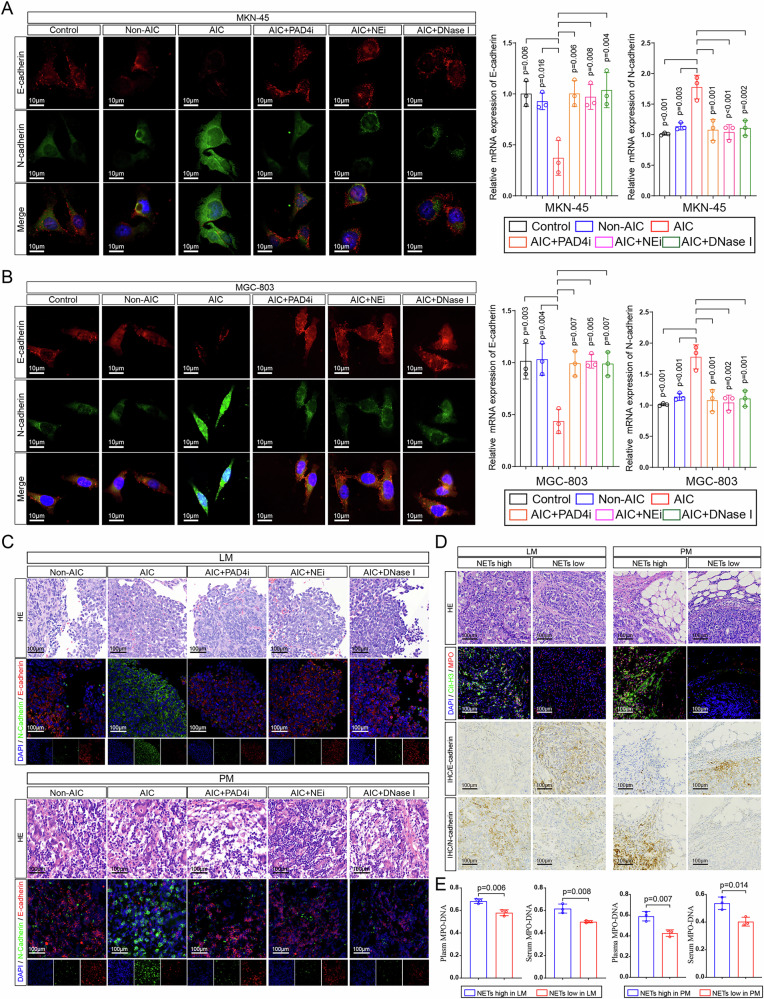


Corrected Figure
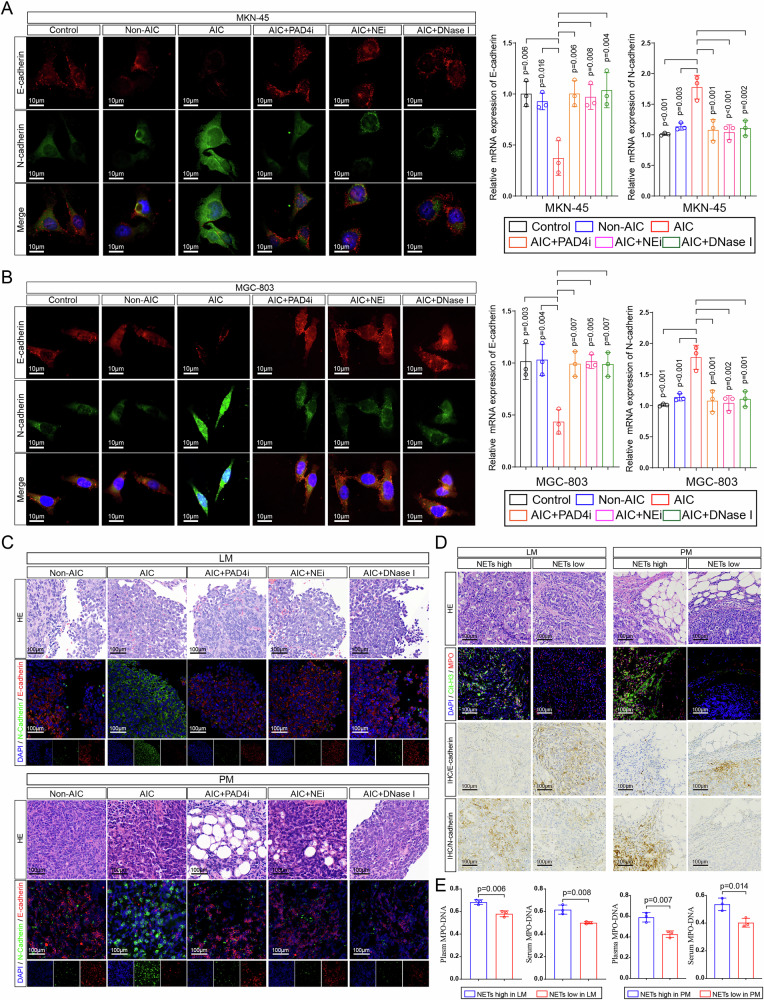


## Supplementary information

Revised Supplementary Information

