# Supplementary material for: Neutrophil extracellular traps promote metastasis in gastric cancer patients with postoperative abdominal infectious complications
Source: Nat Commun. 2022 Feb 23;13:1017. doi: 10.1038/s41467-022-28492-5 (PMC8866499; doi:10.1038/s41467-022-28492-5)
Supplement: Supplementary file 2 — Reporting Summary [file 41467_2022_28492_MOESM2_ESM.pdf]

nature research

Corresponding author(s): Gang Zhao

Last updated by author(s): Jan 1, 2022

Reporting Summary

Nature Research wishes to improve the reproducibility of the work that we publish. This form provides structure for consistency and transparency in reporting. For further information on Nature Research policies, see our Editorial Policies and the Editorial Policy Checklist.

Statistics

For all statistical analyses, confirm that the following items are present in the figure legend, table legend, main text, or Methods section.

n/a	Confirmed	
☐	☒	The exact sample size (*n*) for each experimental group/condition, given as a discrete number and unit of measurement
☐	☒	A statement on whether measurements were taken from distinct samples or whether the same sample was measured repeatedly
☐	☒	The statistical test(s) used AND whether they are one- or two-sided<br>*Only common tests should be described solely by name; describe more complex techniques in the Methods section.*
☒	☐	A description of all covariates tested
☐	☒	A description of any assumptions or corrections, such as tests of normality and adjustment for multiple comparisons
☐	☒	A full description of the statistical parameters including central tendency (e.g. means) or other basic estimates (e.g. regression coefficient) AND variation (e.g. standard deviation) or associated estimates of uncertainty (e.g. confidence intervals)
☐	☒	For null hypothesis testing, the test statistic (e.g. *F*, *t*, *r*) with confidence intervals, effect sizes, degrees of freedom and *P* value noted<br>*Give P values as exact values whenever suitable.*
☒	☐	For Bayesian analysis, information on the choice of priors and Markov chain Monte Carlo settings
☒	☐	For hierarchical and complex designs, identification of the appropriate level for tests and full reporting of outcomes
☒	☐	Estimates of effect sizes (e.g. Cohen's *d*, Pearson's *r*), indicating how they were calculated

Our web collection on statistics for biologists contains articles on many of the points above.

Software and code

Policy information about availability of computer code

Data collection	no software was used
Data analysis	Graphpad Prism 8.0 , Photoshop 5.0, SPSS 23.0, casewiewer 3.0, FlowJo Software version 10.4.2.

For manuscripts utilizing custom algorithms or software that are central to the research but not yet described in published literature, software must be made available to editors and reviewers. We strongly encourage code deposition in a community repository (e.g. GitHub). See the Nature Research guidelines for submitting code & software for further information.

Data

Policy information about availability of data

All manuscripts must include a data availability statement. This statement should provide the following information, where applicable:

- Accession codes, unique identifiers, or web links for publicly available datasets
- A list of figures that have associated raw data
- A description of any restrictions on data availability

The authors declare that all the other data supporting the findings of this study are available within the article and its supplementary information files and from the corresponding author on reasonable request. Specifically, the source data of Figure 1A, B, D, F, Figure 2A, B, C, D, Figure 3A, B, D, Figure 4A, B, C, D, E, F, Figure 5B, C, D, Figure 6D, F, H, J, Supplement figure 1A, B ,C , Supplement figure 2D, E, Supplement figure 3A, B, D, F, G, Supplement figure 4C, D, E, Supplement figure 5E, Supplement figure 6A, B, C, D, F, Supplement figure 7C, D, E are provided as a Source Data file.

Field-specific reporting

Please select the one below that is the best fit for your research. If you are not sure, read the appropriate sections before making your selection.

- [x] Life sciences
- [ ] Behavioural & social sciences
- [ ] Ecological, evolutionary & environmental sciences

For a reference copy of the document with all sections, see nature.com/documents/nr-reporting-summary-flat.pdf

Life sciences study design

All studies must disclose on these points even when the disclosure is negative.

Sample size	The sample size of GC patients cohort was determined by the number of tumor samples and clinic information available in our database. Sample size and number of independent experiments are always clearly stated in the figure legend or in the Methods section and are repeated three or more times independent for statistical analyses. Sample size was not predetermined with statistical methods, but based on expected effect size and variability within the sample, as well as cost and feasibility of experiments
Data exclusions	No data were excluded from analysis.
Replication	Experiments in the article are reliably produced, replication were described in the figure legends
Randomization	experimental samples/ or animals were randomly allocated to experimental groups.
Blinding	Investigators were blinding to group allocation for animals experiments. Also, quantification of tumor parameters by histological analyses was performed blindingly. For cells experiments, investigators were not blinded in performing experiments as they needed to know the different treatments. Where possible, researchers were blinded during data analysis.

Reporting for specific materials, systems and methods

We require information from authors about some types of materials, experimental systems and methods used in many studies. Here, indicate whether each material, system or method listed is relevant to your study. If you are not sure if a list item applies to your research, read the appropriate section before selecting a response.

Materials & experimental systems

n/a	Involved in the study	
[ ]	[x]	Antibodies
[ ]	[x]	Eukaryotic cell lines
[x]	[ ]	Palaeontology and archaeology
[ ]	[x]	Animals and other organisms
[ ]	[x]	Human research participants
[ ]	[x]	Clinical data
[x]	[ ]	Dual use research of concern

Methods

n/a	Involved in the study	
[x]	[ ]	ChIP-seq
[x]	[ ]	Flow cytometry
[x]	[ ]	MRI-based neuroimaging

Antibodies

Antibodies used	PAD4i (10 µM, abcam, ab223598), NEi (5 µM, abcam, ab142369) , anti-Cit-H3 (1:100, Abcam, ab5103), anti-MPO (10 µg/ml, R&D, AF3667), anti-TGF-β1(1:100, Thermo Fisher, MA1-21595; 1:200, Servicebio, GB14154), anti-E-cadherin (1:400, Servicebio, GB11082), anti-N-cadherin (1:500, Servicebio, GB12135), anti-p-Smad2 (1:300, Thermo Fisher, 44-244G), fluorochrome-conjugated secondary antibodies (1:400, Invitrogen; 1:500, Servicebio), DAPI (2 µg/mL, Servicebio, G1012), Hochest 33258 (1µg/ml, Thermo Fisher, H1398), Sytox green (1µM, Thermo Fisher, R37109), anti-MPO monoclonal antibody (R&D, AF3667), TGF-β1 (abcam, ab100647; MultiSciences, 70-EK981-96), IL-8 (MultiSciences, 70-EK108HS-96) , E-cadherin (1:800, Servicebio, GB11082), N-cadherin(1:800, Servicebio, GB11135), Ki67(1:800, Servicebio, GB111499), E-cadherin (1:2000, Abcam, ab40772), N-cadherin(1:2000, Abcam, ab18203), p-Smad2(1:2000, Abcam, ab53100), Smad2(1:2000, Abcam, ab40855), p-Smad3(1:2000, Abcam, ab52903), Smad3(1:2000, Abcam, ab84177), Lamin B1(1:2000, Abcam, ab133741)
Validation	Antibody validations were performed as described on the manufacturers' websites and were supported by multiple publications

Eukaryotic cell lines

Policy information about cell lines

Cell line source(s)	MKN-45, MGC-803 and AGS were purchased from ATCC
Authentication	MKN-45, MGC-803 and AGS cell lines were validated using short tandem repeat (STR) profiling

Mycoplasma contamination	All cell lines were tested to be mycoplasma negative
Commonly misidentified lines<br>(See ICLAC register)	No cell lines used in this study were found in the database of commonly misidentified cell lines that is maintained by ICLAC and NCBI Biosample

Animals and other organisms

Policy information about studies involving animals; ARRIVE guidelines recommended for reporting animal research

Laboratory animals	Eight-week-old female athymic BALB/c nude mice were purchased from Shanghai SLAC Laboratory Animal Co., Ltd. with a weight between 20g and 25g.Animals were housed in Shanghai Genechem SPF animal facility, in temperatures 20-22, خ humidity 30-70% and a 12-hour light/12-hour dark cycle
Wild animals	No wild animals were used in this study
Field-collected samples	No field-collected samples were used in this study
Ethics oversight	The nude mice experiments were approved by Renji Hospital Animal Care and Use Committee

Note that full information on the approval of the study protocol must also be provided in the manuscript.

Human research participants

Policy information about studies involving human research participants

Population characteristics	The detailed participants characteristics such as age, gender and current diagnosis are showed in the study protocol part of the supplementary information file.
Recruitment	All the clinical data were from our medical records. All samples used in our research were from our gastrointestinal department.
Ethics oversight	The Ethics Committees of Renji Hospital affiliated to Shanghai Jiao Tong University School of Medicine approved the study protocols, and written informed consent was obtained from all subjects in this study. All the research was carried out in accordance with the provisions of the Helsinki Declaration of 1975.

Note that full information on the approval of the study protocol must also be provided in the manuscript.

Clinical data

Policy information about clinical studies

All manuscripts should comply with the ICMJEguidelines for publication of clinical research and a completedCONSORT checklist must be included with all submissions.

Clinical trial registration	ChiCTR-PIC-17012358
Study protocol	Study protocol<br><br>Abstract<br><br>Title Standardized records of postoperative complications in patients with gastric cancer and their prognostic implications<br>version/date 1.0/2018-08-03<br>organization Department of Gastrointestinal Surgery, Ren Ji Hospital, School of Medicine, Shanghai Jiao Tong University, Shanghai, P.R. China<br>Principal investigator Zizhen Zhang<br>Study purpose To evaluate and calculate postoperative complications after radical gastrectomy and the influence on prognosis for gastric cancer patients<br>Sample size 1500 cases<br>Study subject Gastric cancer patients<br>Study design Cohort observational study<br>Inclusion criteria 1. Age between 18 to 75 years old; 2. Primary gastric adenocarcinoma confirmed pathologically by endoscopic biopsy; 3. Ear or locally advanced tumor gastric cancer; 4. No distant metastasis, no direct invasion of pancreas, spleen or other organs nearby in the preoperative examinations; 5. Performance status of 0 or 1 on ECOG (Eastern Cooperative Oncology Group) scale; 6. ASA (American Society of Anesthesiology) class I to III; 7. Written informed consent.<br>Exclusion criteria 1. Pregnant and lactating women; 2. Suffering from severe mental disorder; 3. History of previous upper abdominal surgery (except for laparoscopic cholecystectomy); 4. History of previous gastric surgery (including ESD/EMR (Endoscopic Submucosal Dissection/Endoscopic Mucosal Resection)for gastric cancer); 5. History of other malignant disease within the past 5 years; 6. History of unstable angina or myocardial infarction within the past 6 months; 7. History of cerebrovascular accident within the past 6 months; 8. History of continuous systematic administration of corticosteroids within 1 month; 9. Requirement of simultaneous surgery for other disease; 10. Emergency surgery due to complication (bleeding, obstruction or perforation) caused by gastric cancer; 11. FEV1 50% of the predicted values.<br>Study execute time from 2018-08-14 To 2019-08-13
Data collection	Medical records of gastric cancer patients from 2010 to 2015 in Renji Hospital affiliated to Shanghai Jiao Tong University School of

Medicine were recruited. Data were collected from 2018-08-14 To 2019-08-13

Outcomes	Primary outcomes: Overall survival<br>Secondary outcomes: 1. Relapse free survival; 2. Postoperative complication rate; 3. Postoperative infectious complication rate; 4. Neutrophils rate and amount; 5. Neutrophil extracellular traps(NETs); 6. NETs related biomarkers.
