# Supplementary material for: Author Correction: Neutrophil extracellular traps promote metastasis in gastric cancer patients with postoperative abdominal infectious complications
Source: Nat Commun. 2025 Mar 10;16:2381. doi: 10.1038/s41467-025-57805-7 (PMC11894055; doi:10.1038/s41467-025-57805-7)

Supplementary Information for

Neutrophil extracellular traps promote metastasis in gastric cancer patients with postoperative abdominal infectious complications

Supplementary Figures

Supplementary figure 1 **Postoperative AIC decrease OS and RFS in locally advanced GC patients undergoing curative surgery.**

Supplementary figure 2 **Neutrophils are isolated from peripheral blood or ascites fluid for NETs.**

Supplementary figure 3 **Coculturing with no NETs-containing neutrophils did not promote proliferation, invasion or migration of GC cells**.

Supplementary figure 4 **The effects of TGF-β1 on EMT, invasion, migration and proliferation of GC cells**.

Supplementary figure 5 **A modified infection model design to mimic postoperative AIC-induced NETs in nude mice.**

Supplementary figure 6 **Cecal puncture without ligation nude mice model design for AIC-induced NETs-GC clusters.**

Supplementary figure 7 **CP (25G) modeling imitates postoperative AIC of GC patients.**

Supplementary Note 1. **Clinical trial study protocol. Main information (translated version of the original study protocol**

Supplementary figure 1

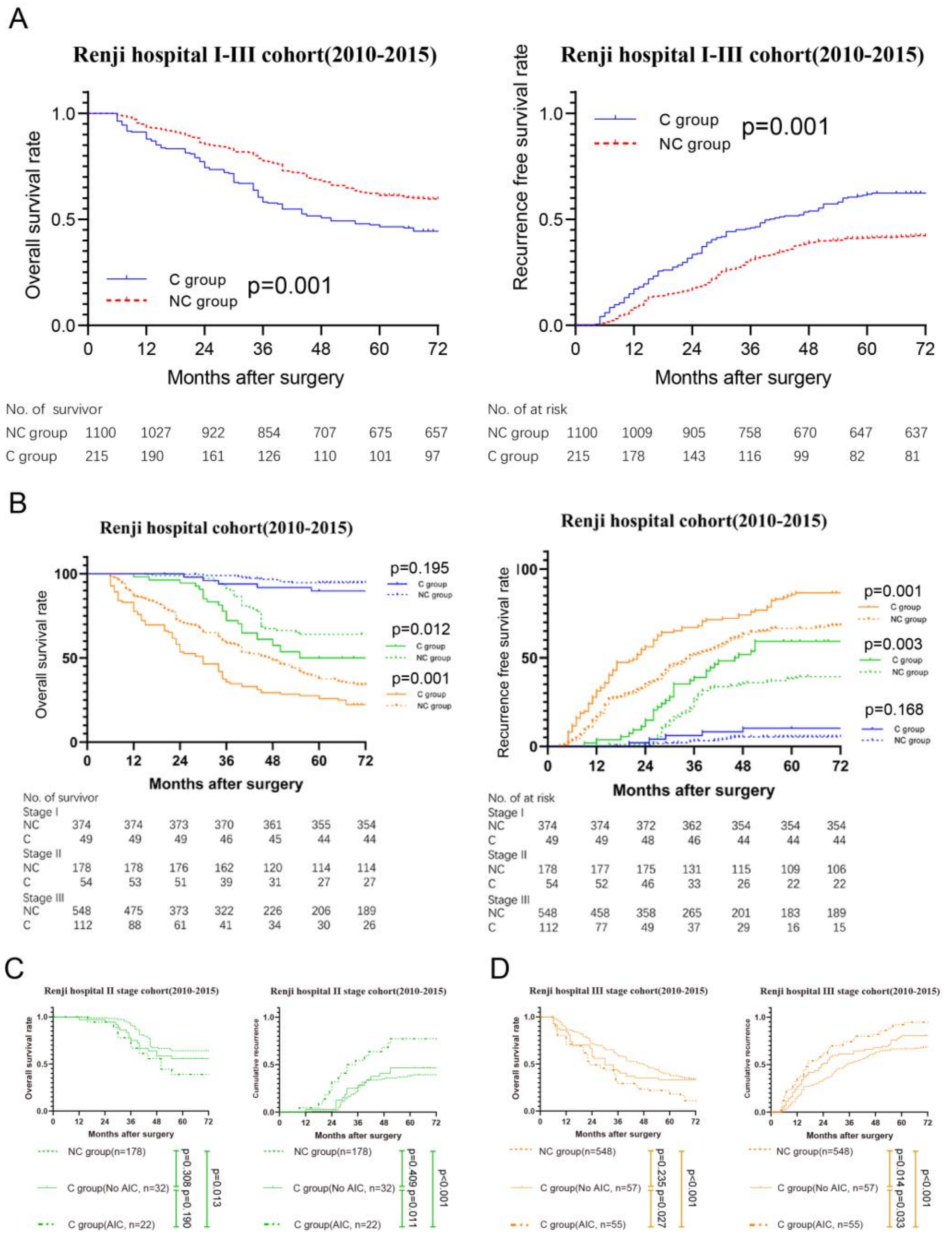

Supplementary figure 1

Postoperative AIC decrease OS and RFS in locally advanced GC patients undergoing curative surgery. (A, B) Kaplan–Meier estimate of OS and RFS in the entire (A) and stratified pStage (B) GC cohorts undergoing curative surgery grouped by complication group (C group) and no complication group (NC group); (C, D) Kaplan–Meier estimate of OS and RFS in pStage II (C) and III (D) GC cohorts undergoing curative surgery among NC, C (No AIC) and C(AIC) groups. Log-rank tests were used in A, B, C, D for OS and RFS comparison. In A and B, $P<0.05$ was considered to indicate a statistically significant difference. In C and D, P values were adjusted for multiple comparisons using the Bonferroni correction and $P<0.017$ (0.05/3) was defined as statistically significant. Source data are provided as a Source Data file.

Supplementary figure 2

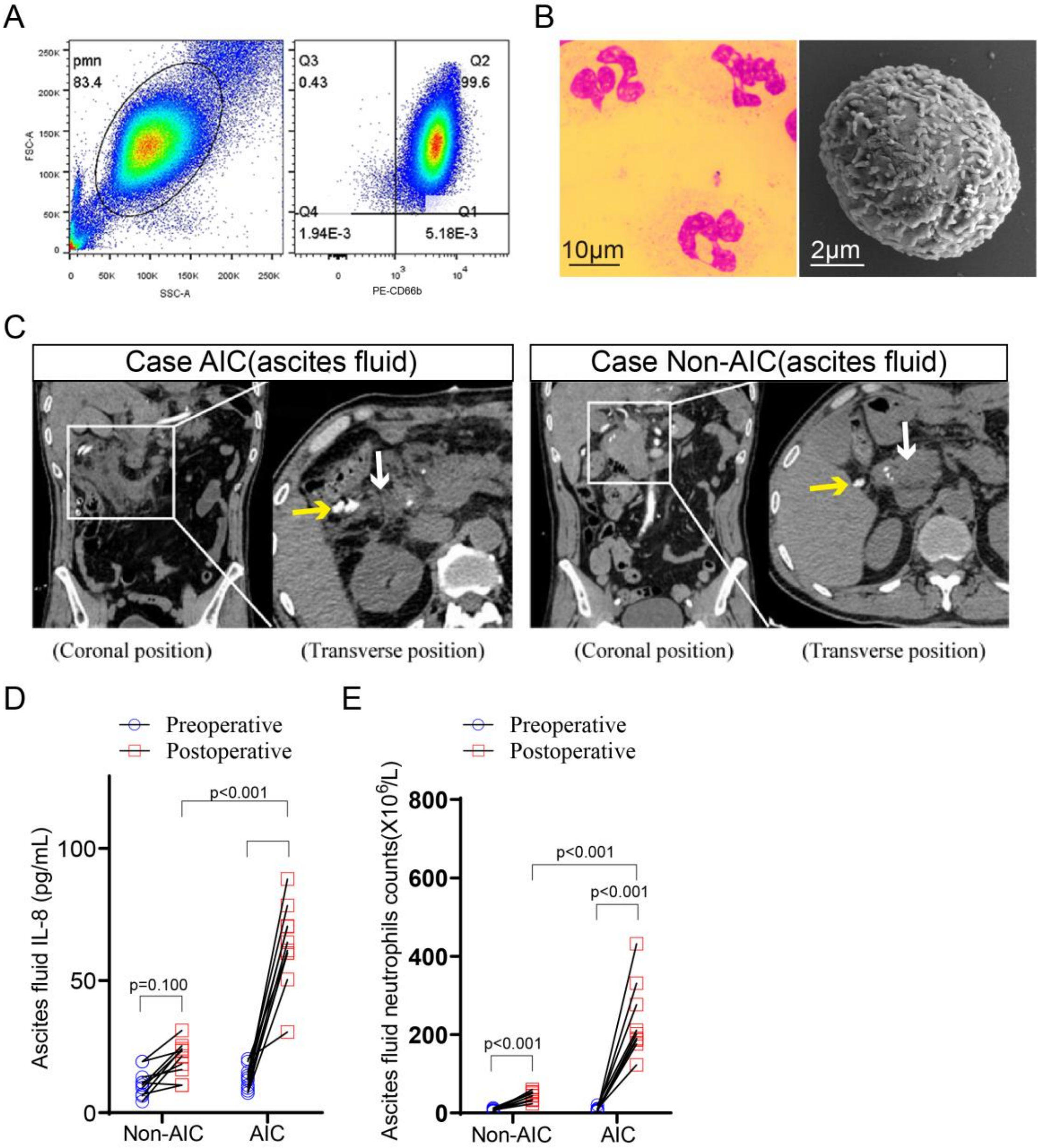

Supplementary figure 2

Neutrophils are isolated from peripheral blood or ascites fluid for NETs. (A) Purity of neutrophils evaluated by flow cytometry, showing forward and side scatter and staining for CD66B-PE in right panel; (B) Isolated neutrophils were observed in optical microscopy and SEM; (C) Representative Computed Tomography (CT) images of postoperative ascites fluid (white arrow) and abdominal drainage(yellow arrow) (n= 10 per group). (D) Preoperative and postoperative ascites fluid IL-8 levels between Non-AIC and AIC groups. (E) Preoperative and postoperative ascites fluid neutrophils counts between Non-AIC and AIC groups. Paired and unpaired Student's t tests were used in D, E (n= 10 per group). B were representative of three biologically independent experiments. Source data are provided as a Source Data file.

Supplementary figure 3

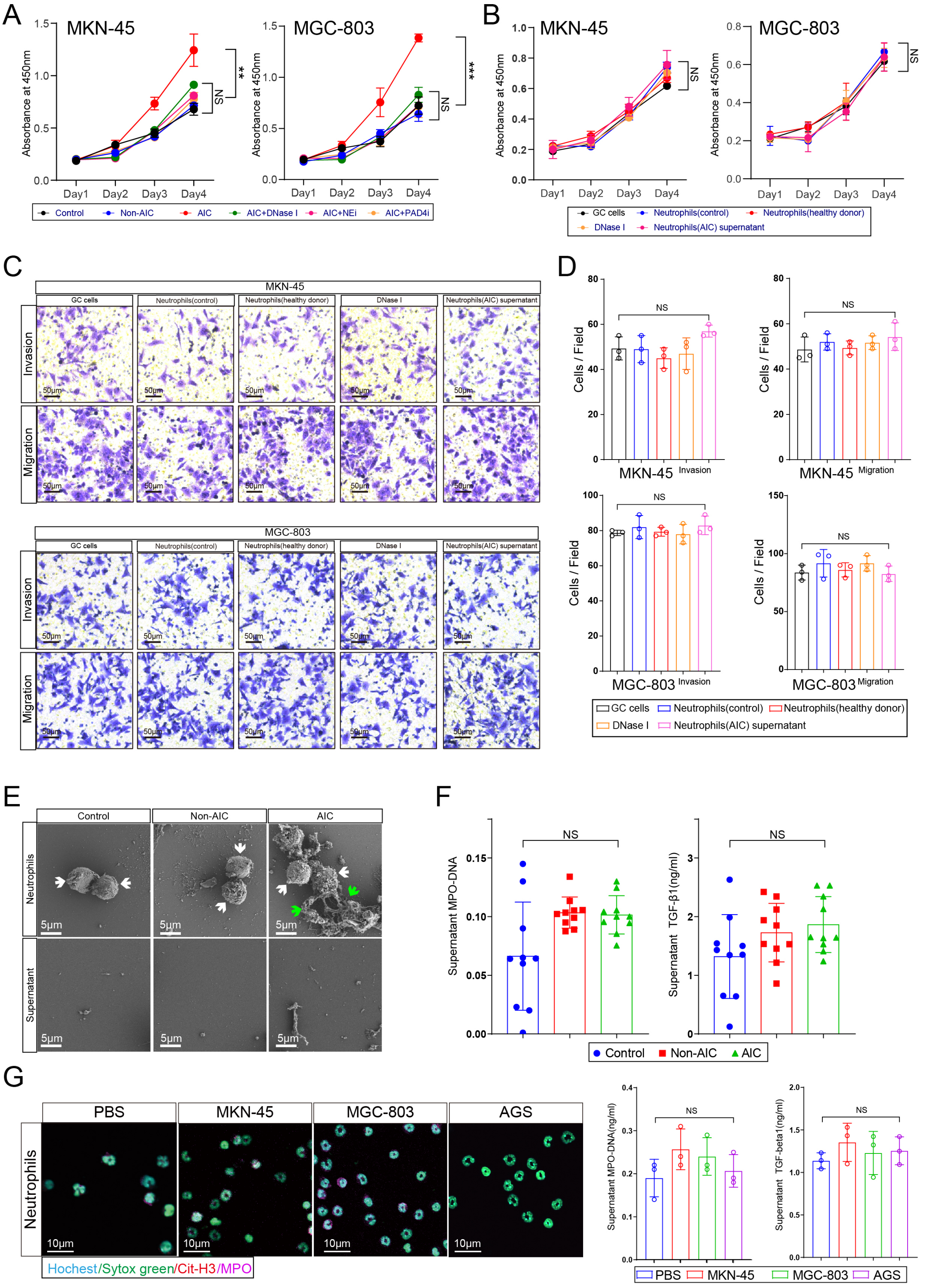

Supplementary figure 3

Coculturing with no NETs-containing neutrophils did not promote proliferation, invasion or migration of GC cells. (A, B) Results of coculturing GC cells with indicated neutrophils or agents for proliferation assay (MKN-45: AIC vs Control, P<0.001. AIC vs Non-AIC, P<0.001. AIC vs AIC+DNase I, P<0.001. AIC vs NEi, P<0.001. AIC vs PAD4i, P=0.001. MGC-803: AIC

vs Control, P<0.001. AIC vs Non-AIC, P<0.001. AIC vs AIC+DNase I, P<0.001. AIC vs NEi, P<0.001. AIC vs PAD4i, P=0.003). (C, D) Transwell Matrigel invasion and migration assays of MKN-45(upper panel) and MGC-803(down panel) cells subjected to indicated neutrophils or agentst; (E) Representative SEM of neutrophils and neutrophils supernatant isolated from control, Non-AIC and AIC groups. green arrows point to extracellular meshes of NETs and white arrows point to neutrophils (n= 10 per group); (F) Supernatant MPO-DNA and TGF-β1 levels in neutrophils isolated from control, Non-AIC and AIC groups; (G) Representative immunofluorescence co-staining of DNA, Cit-H3 and MPO to assess NETs formation in the neutrophils coculturing with GC cells (MKN-45, MGC-803 and AGS) and their supernatant MPO-DNA and TGF-β1 levels were compared; Data represent the mean ± S.D. in A, B, D, G (n= 3 biologically independent experiments) and F (n= 10 per group); one-way ANOVA with Tukey test was used in A, B, D, F, G. Source data are provided as a Source Data file.

Supplementary figure 4

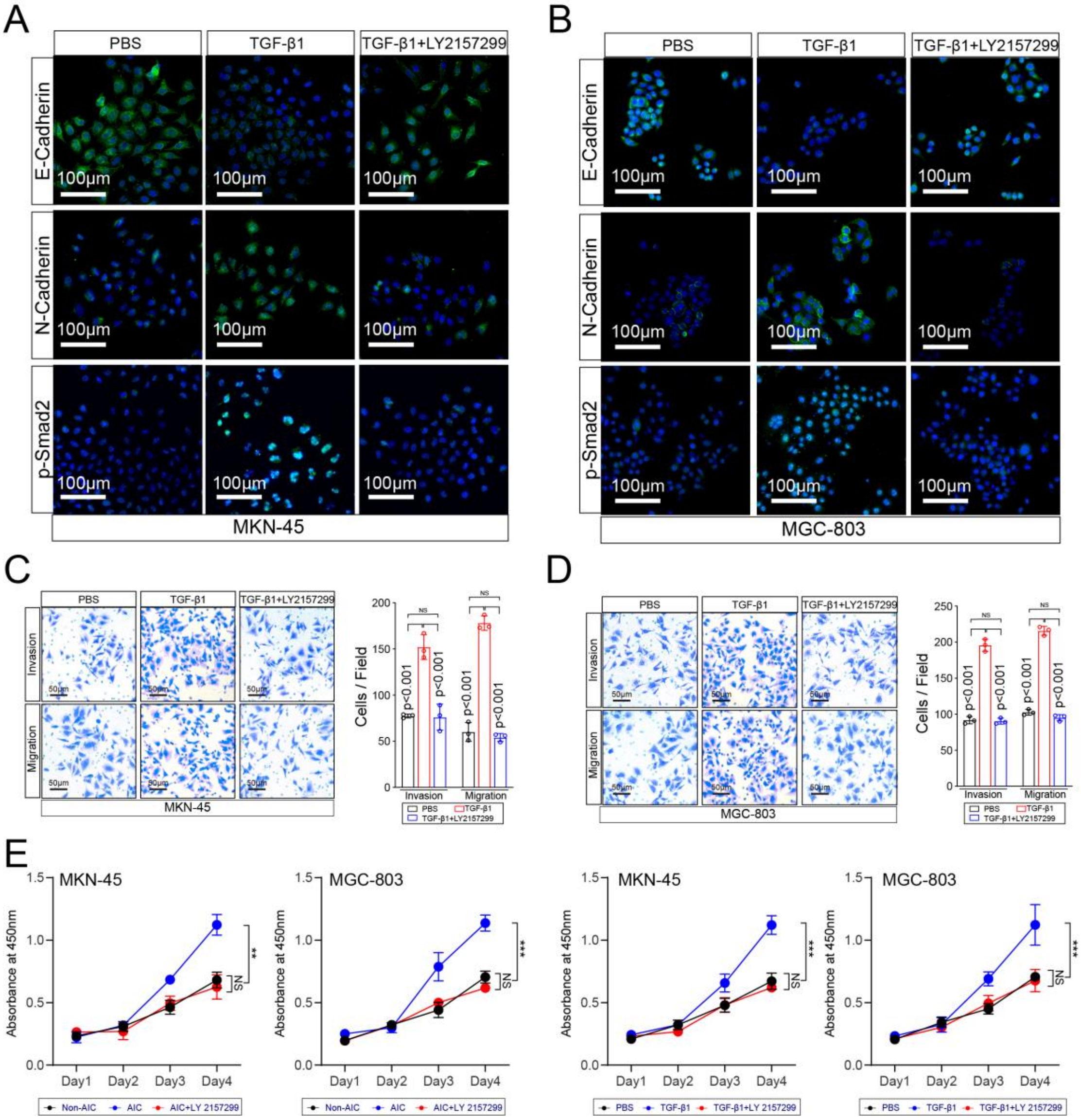

Supplementary figure 4

The effects of TGF-β1 on EMT, invasion, migration and proliferation of GC cells. (A, B) Representative immunofluorescence staining showing EMT markers (E-cadherin and N-cadherin) and TGF-β signaling pathway (p-Smad2) changes upon TGF-β1 and TGF-β inhibitor LY 2157299 treatment in MKN-45 (A) and MGC-803 (B) cells (n= 3 biologically independent experiments); (C, D) Transwell Matrigel invasion and migration assays of MKN-45(C) and MGC-803(D) cells subjected to TGF-β1 and TGF-β inhibitor LY 2157299 treatment. (E) Proliferation assay of MKN-45 and MGC-803 cells subjected to indicated treatment (MKN-45: AIC vs Non-AIC, P=0.002. AIC vs AIC+ LY 2157299, P<0.001. MGC-803: AIC vs Non-AIC, P<0.001. AIC vs AIC+ LY 2157299, P<0.001. MKN-45: TGF-β1 vs PBS, P<0.001. TGF-β1vs TGF-β1+ LY 2157299, P<0.001. MGC-803: AIC vs Non-AIC, P=0.008. AIC vs AIC+ LY 2157299, P=0.006.). Data represent the mean ± S.D. in C, D, E (n= 3 biologically independent experiments); one-way ANOVA with Tukey test was used in C, D, E. Source data are provided as a Source Data file.

Supplementary figure 5

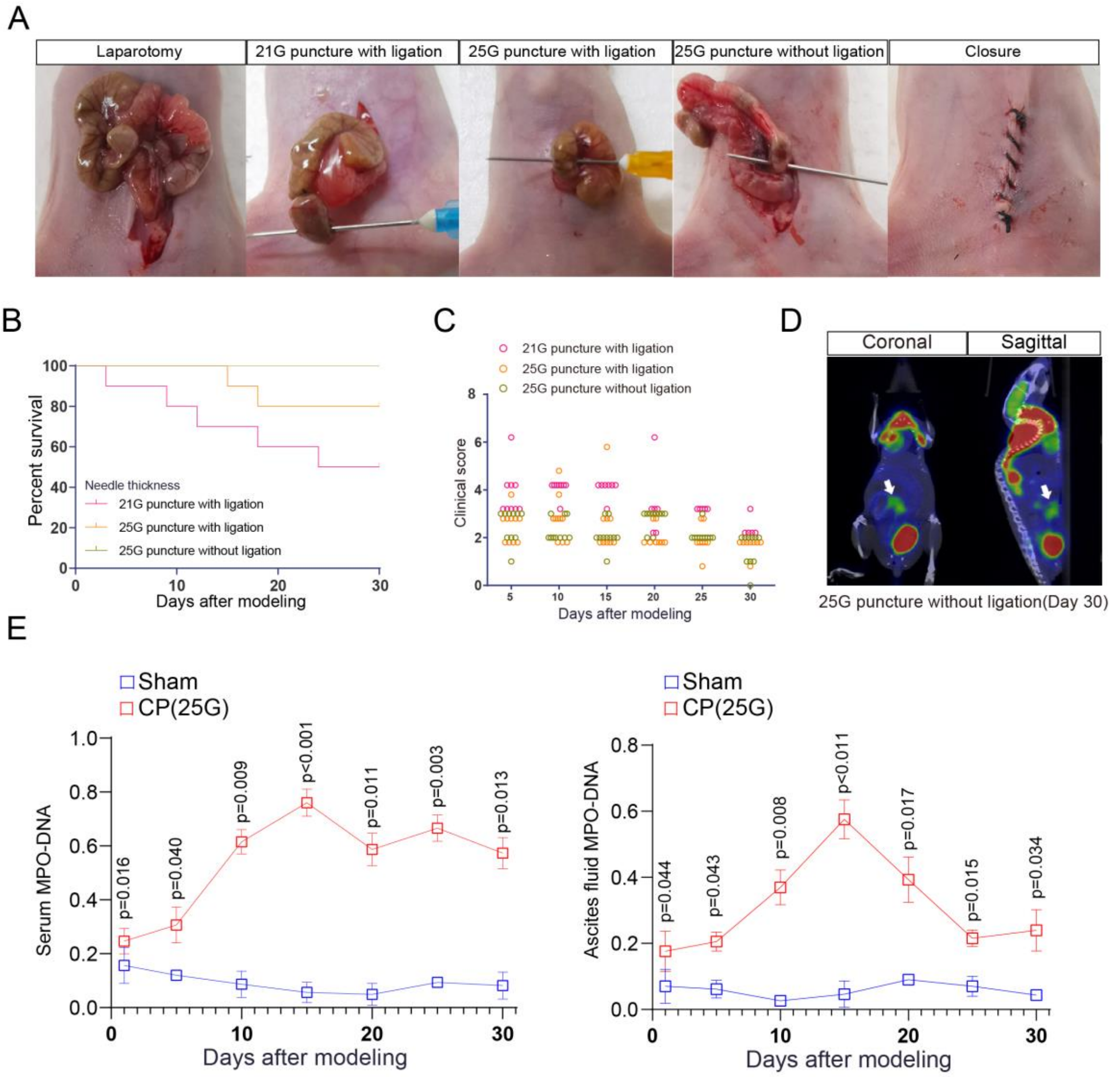

Supplementary figure 5

A modified infection model design to mimic postoperative AIC-induced NETs in nude mice. (A) The procedure of CP with or without ligation in the nude mice; (B, C) The survival curves (B) and sepsis clinical scores (C) of nude mice undergoing indicated modeling (n= 5 per group); (D) Representative images of PET/CT to determine postoperative AIC. White arrows point to the infectious site in abdominal cavity; (E) Serum and ascites fluid MPO-DNA level in nude mice during post-modeling 30 days; Data represent the mean ± S.D. in E (n= 3 per group), one-way ANOVA with Tukey test was used in E. Source data are provided as a Source Data file.

Supplementary figure 6

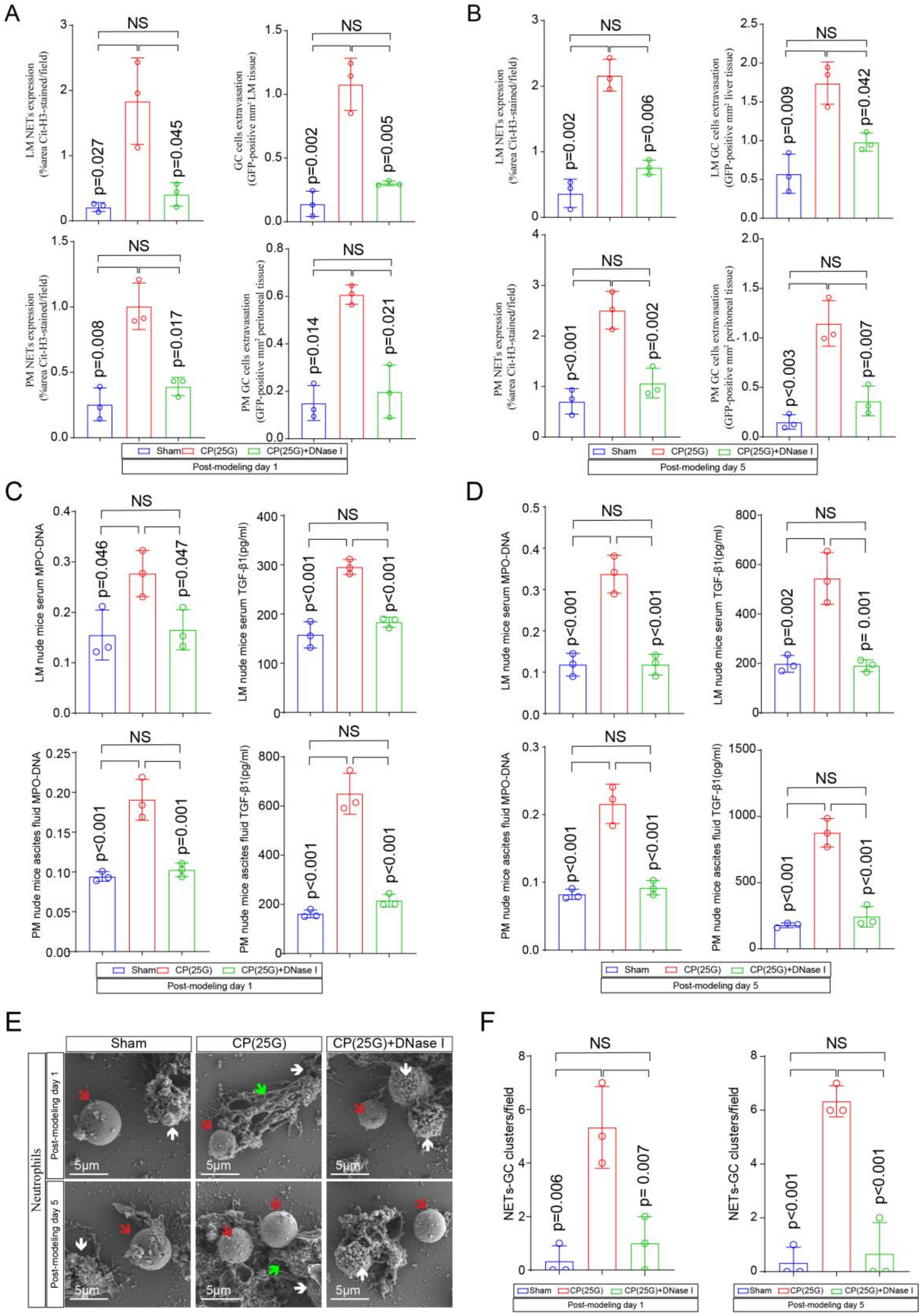

Supplementary figure 6

Cecal puncture without ligation nude mice model design for AIC-induced NETs-GC clusters. (A) quantification of NETs, GC cells extravasation in the liver of LM nude mice and GC cells implantation in the peritoneum of PM nude mice at post-modeling day 1; (B) quantification of

NETs, GC cells extravasation in the liver of LM nude mice and GC cells implantation in the peritoneum of PM nude mice at post-modeling day 5; (C) Serum and ascites fluid MPO-DNA and TGF-β1 levels collected from LM and PM nude mice at post-modeling day 1; (D) Serum and ascites fluid MPO-DNA and TGF-β1 levels collected from LM and PM nude mice at post-modeling day 5; (E, F) Representative SEM (E) and quantification (F) of NETs-GC clusters in the lower chamber medium after neutrophils (isolated from modeling nude mice) and MKN-45 cells were subjected to upper chamber simultaneously for NETs-GC clusters transwell assay. Data represent the mean ± S.D. in A, B, C, D, F (n= 3 per group), one-way ANOVA with Tukey test was used in A, B, C, D, F. Source data are provided as a Source Data file.

Supplementary figure 7

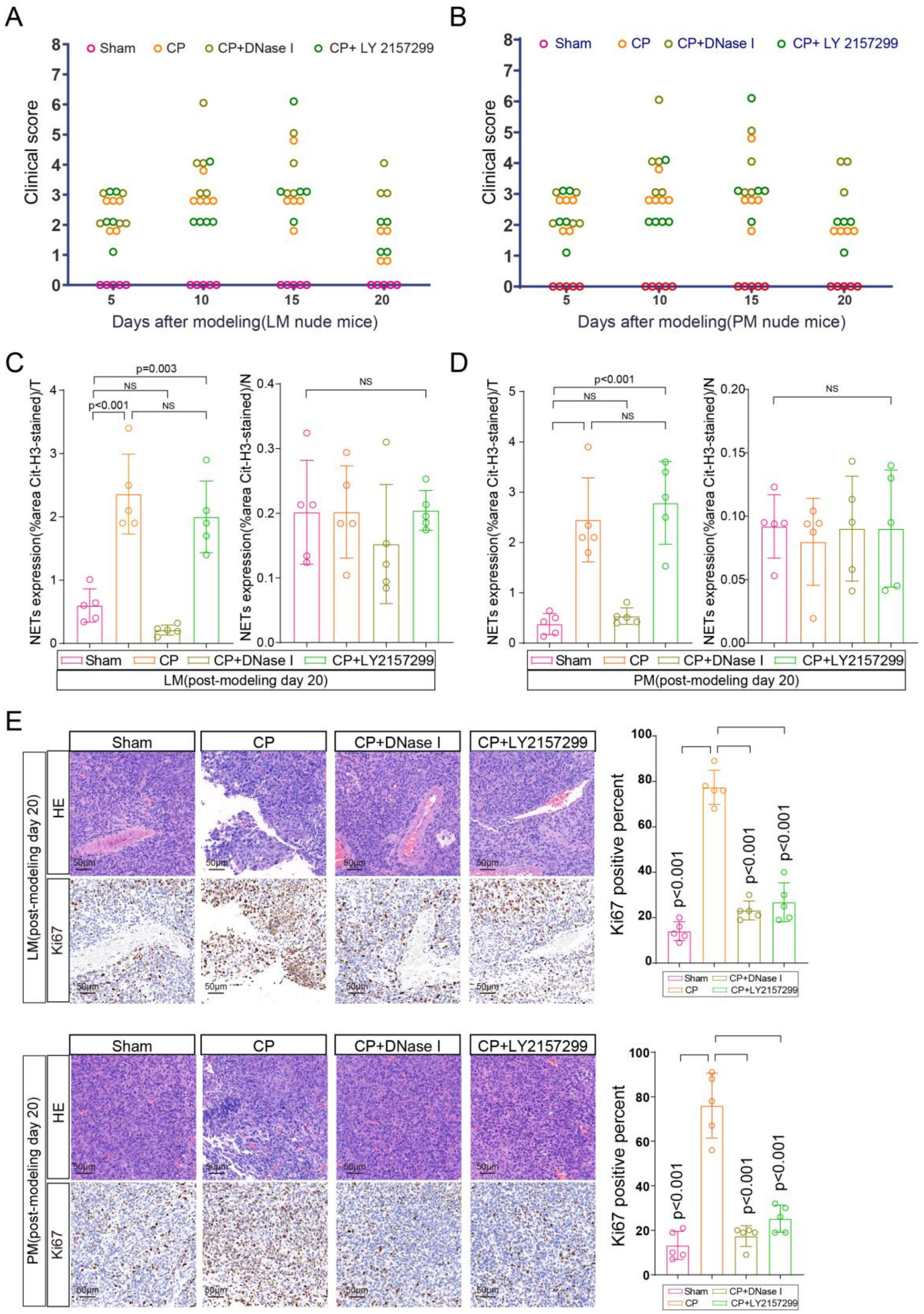

Supplementary figure 7

CP (25G) modeling imitates postoperative AIC of GC patients. (A, B) The clinical scores for sepsis evaluation every 5 days in LM (A) and PM (B) nude mice as indicated modeling (n=5 per group); (C) Quantification of NETs in T (tumor) and N (normal) tissue in the liver of LM nude mice

at post-modeling day 20; (D) Quantification of NETs in T (tumor) and N (normal) tissue in the peritoneum of PM nude mice at post-modeling day 20; (E) Representative HE and IHC of proliferation marker Ki67 with quantification in the metastatic lesions of LM nude mice (upper panel) and PM nude mice (down panel) at post-modeling day 20. Data represent the mean ± S.D. in C, D, E (n= 5 per group), one-way ANOVA with Tukey test was used in C, D, E. Source data are provided as a Source Data file.

Supplementary Note 1.

Clinical trial study protocol
Main information (translated version of the original study protocol

Title	Standardized records of postoperative complications in patients with gastric cancer and their prognostic implications
version/date	1.0/2018-08-03
organization	Department of Gastrointestinal Surgery, Ren Ji Hospital, School of Medicine, Shanghai Jiao Tong University, Shanghai, P.R. China
Principal investigator	Zizhen Zhang
Study purpose	To evaluate and calculate postoperative complications after radical gastrectomy and the influence on prognosis for gastric cancer patients
Sample size	1500 cases
Study subject	Gastric cancer patients
Study design	Cohort observational study
Inclusion criteria	1. Age between 18 to 75 years old; 2. Primary gastric adenocarcinoma confirmed pathologically by endoscopic biopsy; 3. Ear or locally advanced tumor gastric cancer; 4. No distant metastasis, no direct invasion of pancreas, spleen or other organs nearby in the preoperative examinations; 5. Performance status of 0 or 1 on ECOG (Eastern Cooperative Oncology Group) scale; 6. ASA (American Society of Anesthesiology) class I to III; 7. Written informed consent.
Exclusion criteria	1. Pregnant and lactating women; 2. Suffering from severe mental disorder; 3. History of previous upper abdominal surgery (except for laparoscopic cholecystectomy); 4. History of previous gastric surgery (including ESD/EMR (Endoscopic Submucosal Dissection/Endoscopic Mucosal Resection)for gastric cancer); 5. History of other malignant disease within the past 5 years; 6. History of unstable angina or myocardial infarction within the past 6 months; 7. History of cerebrovascular accident within the past 6 months; 8. History of continuous systematic administration of corticosteroids within 1 month; 9. Requirement of simultaneous surgery for other disease; 10. Emergency surgery due to complication (bleeding, obstruction or perforation) caused by gastric cancer; 11. FEV1 < 50% of the predicted values.
Study execute time	From 2018-08-14 To 2019-08-13
Primary outcomes:	Overall survival

Secondary outcomes:	1. Relapse free survival; 2. Postoperative complication rate; 3. Postoperative infectious complication rate; 4. Neutrophils rate and amount; 5. Neutrophil extracellular traps(NETs); 6. NETs related biomarkers.

Study protocol

	Screen	Recruitment	surgery	discharge	Follow up
					Postoperative X months
Time frame	Day -7	Day -1	Day 0	Day 7+	Month X
Window phase	±3Day		±1 Day	±3 Day	±7 Day
informed consent form signature		√			
inclusion/exclusion criterion		√			
Physical examination	√			√	√
Blood sample collection		√		√√√	√
Chest X ray	√				√
Gastroscopy and pathological confirmation	√				
Abdominal enhanced CT	√				√
Pathological documentation	√			√	

√ check point;

Perioperative management:

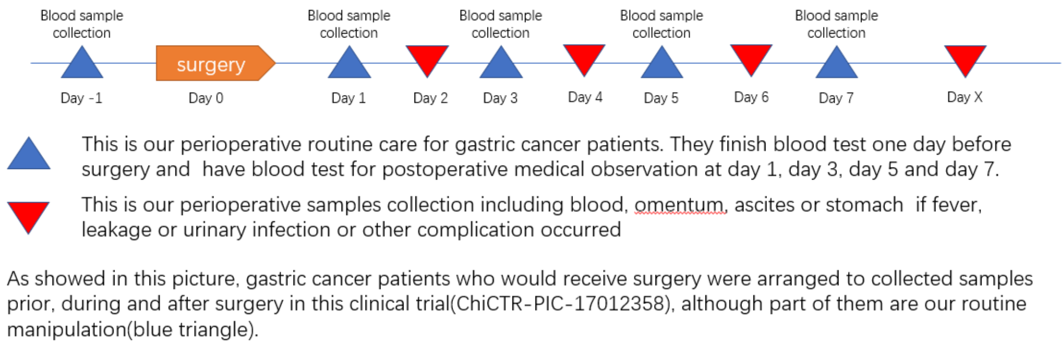

Supplement: Supplementary file 1 — Revised Supplementary Information [file 41467_2025_57805_MOESM1_ESM.pdf]
